# Meta-analysis of the association between the inflammatory potential of diet and colorectal cancer risk

**DOI:** 10.18632/oncotarget.19233

**Published:** 2017-07-14

**Authors:** Yu Fan, Xin Jin, Changfeng Man, Zhenjun Gao, Xiaoyan Wang

**Affiliations:** ^1^ Institute of Molecular Biology and Translational Medicine, The Affiliated People's Hospital, Jiangsu University, Zhenjiang, Jiangsu, 212002, China; ^2^ Department of Digestive Disease, Central Hospital of Shanghai Qingpu District, Shanghai, 201700, China; ^3^ Department of Digestive Disease, The First People's Hospital of Suqian City, Suqian, Jiangsu, 223800, China

**Keywords:** dietary inflammatory index, colorectal cancer, meta-analysis, systematic review

## Abstract

**Objectives:**

The inflammatory potential of diet has been inconsistently linked to colorectal cancer (CRC) risk. This meta-analysis aimed to evaluate the association of the inflammatory potential of diet, as estimated by the dietary inflammatory index (DII) score, with CRC risk.

**Materials and Methods:**

The PubMed and Embase databases were searched for relevant studies from inception to February 2017. All cohort and case–control studies investigating the association of the DII score with CRC risk were selected.

**Results:**

Four prospective cohorts and four case–control studies, which enrolled a total of 880,380 participants, were included. The pooled adjusted risk ratio (RR) of CRC for the highest DII score versus the lowest category was 1.43 (95% confidence interval [CI]: 1.26–1.62). When stratified by study design, the RRs for the case–control and cohort studies were 1.27 (95% CI: 1.16–1.38) and 1.81 (95% CI: 1.48–2.22), respectively. Subgroup analysis showed that individuals with the highest category of DII score were independently associated with CRC risk in men (RR=1.51; 95% CI: 1.29–1.76), women (RR=1.25; 95% CI: 1.10–1.41), colon cancer (RR=1.39; 95% CI: 1.19–1.62), and rectal cancer (RR=1.32; 95% CI: 1.01–1.74). However, the pooled RR was 1.07 (95% CI: 0.87–1.31) for rectal cancer among the prospective cohort studies.

**Conclusions:**

As estimated by a high DII score, pro-inflammatory diet is independently associated with increased CRC risk. This finding confirms that low inflammatory potential diet may reduce CRC risk. However, the gender- and cancer site-specific associations of the DII score with CRC risk need to be further investigated.

## INTRODUCTION

Colorectal cancer (CRC) is the third most common cancer in males and the second in females, with an estimated 1.4 million cases occurring in 2012 [1]. Lifestyle, genetic, and environmental factors have been associated with increased CRC risk. Chronic inflammation plays a central role in carcinogenesis [2, 3]. Diet components can reduce cancer risk by suppressing chronic inflammation [4]. Increased consumption of red and processed meats is strongly associated with increased CRC risk, whereas high fruit/vegetable intake is inversely linked to CRC [5]. Individuals who frequently consume vegetables, fruits, whole grains, nuts, seeds, healthy oils, and fish may possess a low risk of inflammation-related diseases [6]. Therefore, modulating the inflammatory potential of diet may reduce CRC risk.

Several dietary indices, such as the Healthy Eating Index [7], Alternate Healthy Eating Index [8], and Dietary Approaches to Stop Hypertension [9], have been used to assess diet quality. However, none of these studies focused on the inflammatory potential of diet. A novel tool known as the dietary inflammation index (DII) is a literature-derived, population-based score that reflects the inflammatory potential of an individual's diet [10, 11]. The DII score distinguishes dietary patterns from the maximal pro-inflammatory components to the maximal anti-inflammatory components. A higher DII score indicates a more pro-inflammatory diet, whereas a lower DII score represents a more anti-inflammatory diet. The inflammatory potential of diet, as estimated by the DII score, could influence CRC [12–19]. However, the gender- or cancer site-specific risk estimates in these studies are inconsistent.

To our knowledge, no previous meta-analysis has addressed this issue. The current meta-analysis aimed to investigate the association between the inflammatory potential of diet, as estimated by the DII score, and CRC risk.

## RESULTS

### Search results and study characteristics

**Figure 1:**
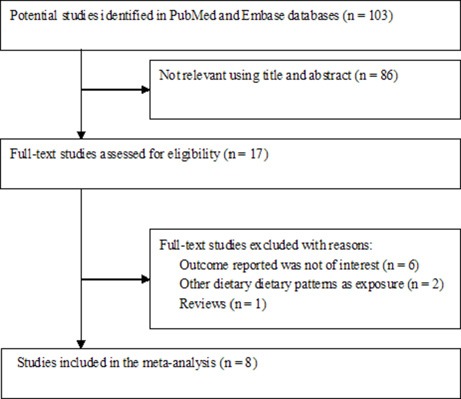
Flow chart of the study selection process

Figure 1 shows the study selection process. A total of 8 eligible studies [12–19] from 103 relevant articles were identified in this meta-analysis. The main characteristics of these studies are summarized in Table 1. Among these 8 studies, 4 used a prospective cohort design [12, 14, 15, 19], whereas the other 4 were case–control studies [13, 16–18]. The sample sizes ranged from 355 to 489,525, with a total number of 880,380 participants. The included studies were published between 2014 and 2017 and performed in the United States [12, 14, 15, 19], France [16], Italy [13], Jordan [18], and Korea [17]. In the prospective cohort studies, the follow-up duration ranged from 9.1 years to 20 years. Two studies [12, 14] enrolled women participants only, whereas others included both men and women participants. The DII score was assessed through validated food frequency questionnaires (FFQs) or dietary history questionnaire. All the included studies achieved 6–8 stars, and their mean Newcastle–Ottawa scale (NOS) score was 7.13.

**Table 1:** Characteristics of studies included in the meta-analysis

Study/year	Country	Study design	Sample size	Source of controls	Median/mean age (years)	% Female	Dietary assessment	DII score comparison	HR or OR (95% CI)	Adjustment for covariates	Follow-up (years)	NOS stars
Shivappa et al. 2014 [12]	USA	Prospective cohort study	37,403 (1,329 colon and 325 rectal cancer)	—	55–69	100%	FFQ (121 items)	Quintile 5 vs. 1; > 1·10 vs.< −2.75	Colorectal cancer 1.20 (1.01–1.43); Colon cancer; 1.19 (0.98–1.45) Rectal cancer; 1.21 (0.81–1.79)	Age, BMI, smoking status, pack-years of smoking, HRT use, education, DM, and total energy intake	19.6	7
Shivappa et al. 2015 [13]	Italy	Case–control	Case:1953; Con:4154	Hospital based	Case 62; Con:58	Case:42.4% Con: 50%	FFQ-derived dietary data	Quintile 5 vs. 1; > 1·22 vs.≤ −1.05	Colorectal cancer 1.55 (1.29–1.85) 1.90 (1.47–2.45) M 1.27 (1.00–1.65) F Colon cancer; 1.39 (1.13–1.71) Rectal cancer; 1.47 (1.14–1.90)	Age, sex, study centre, education,, BMI, alcohol drinking, PA, history of CRC, and energy intake	—	7
Tabung et al. 2015 [14]	USA	Prospective cohort study	152,536 (1,559 colon and 361 rectal)	—	50–79	100%	FFQ (122 items)	Quintile 5 vs. 1; > 1·953 vs. < −3.14	Colorectal cancer 1.22 (1.05–1.43); Colon cancer; 1.23 (1.03–1.46) Rectal cancer; 1.20 (0.84–1.72)	Age, total energy intake, BMI, race/ethnicity, PA, education, smoking, family history of CRC, hypertension, DM, arthritis, history of colonoscopy or occult blood tests, NSAID use, estrogen and/or progesterone use, and different trial arms	11.3	7
Wirth et al. 2015 [15]	USA	Prospective cohort study	489,422 (6,944 cases)	—	62.0 ± 5.4	40.3%	FFQ (124 items)	Quartile 4 vs. 1; > 3.25 vs. < −0.59	Colorectal cancer 1.40 (1.28–1.53) 1.44 (1.29–1.61) M 1.12 (0.95–1.31) F Rectal cancer; 0.91 (0.67–1.25)	Age, smoking status, BMI, self-reported diabetes, energy intake, PA, marital status, education.	9.1	8
Zamora-Ros et al.2015 [16]	France	Case–control	Case:424; Con:401	Hospital based	65.8 ± 12	56%	Dietary history questionnaire	Quartile 4 vs. 1; > 3.05 vs.< −0.73	Colorectal cancer 1.65 (1.05–2.60); Colon cancer; 2.24 (1.33–3.77); Rectal cancer; 1.12 (0.61–2.06)	Age, sex, total energy intake, BMI, first-degree family history of CRC, PA, tobacco consumption, and medication use	—	7
Cho et al. 2016 [17]	Korea	Case–control	Case:923; Con:1846	Health check-up	Case:56.6; Con: 56.1	32.3%	Semi-quantitative FFQ (116 items)	Tertile 3 vs. 1; ≥ 1.76 vs. ≤ -0.28	Colorectal cancer 2.16 (1.71–2.73) 1.72 (1.30–2.28) M 2.50 (1.64–3.82) F Colon cancer; 2.05 (1.53–2.74) Rectal cancer; 2.23 (1.66–3.00)	Age, sex, BMI, education, family history of CRC, PA, and total calorie intake.	—	7
Shivappa et al. 2017 [18]	Jordan	Case–control	Case:153; Con:202	Hospital personnel, outpatients, and visitors	Case:51.6; Con: 53.8	Case:52.3% Con: 49%	FFQ (111 items)	Tertile 3 vs. 1; > 2.18 vs. ≤ −1.38	Colorectal cancer 2.13 (1.23– 3.72)	Age, sex, education, PA, BMI, smoking, family history of CRC.	—	6
Harmon et al. 2017 [19]	USA	Prospective cohort study	190,963 (3372 colon, 981 rectum, and 35 with both)	—	45–75	55%	FFQ(180 items)	Quartile 4 vs. 1; > −0.52 vs.< −3.66	Colorectal cancer 1.21 (1.11–1.32) 1.28 (1.13–1.45) M 1.16 (1.02–1.33) F Colon cancer; 1.20 (1.09–1.33)	Age, sex, race, DM, asthma, heart attack, use of supplements, smoking, family history of colon cancer, education, use of HRT or aspirin	20	8

Abbreviations: M, male; F, female; OR, odds ratio; HR, hazard ratio; CI, confidence interval; BMI, body mass index; CRC, colorectal cancer; DM, diabete mellitus; SBP, systolic blood pressure; DBP, diastolic blood pressure; DII, dietary inflammatory index; FFQ, food frequency questionnaire; HRT, hormone replacement therapy; PA, physical activity.

### DII score and CRC risk

**Figure 2:**
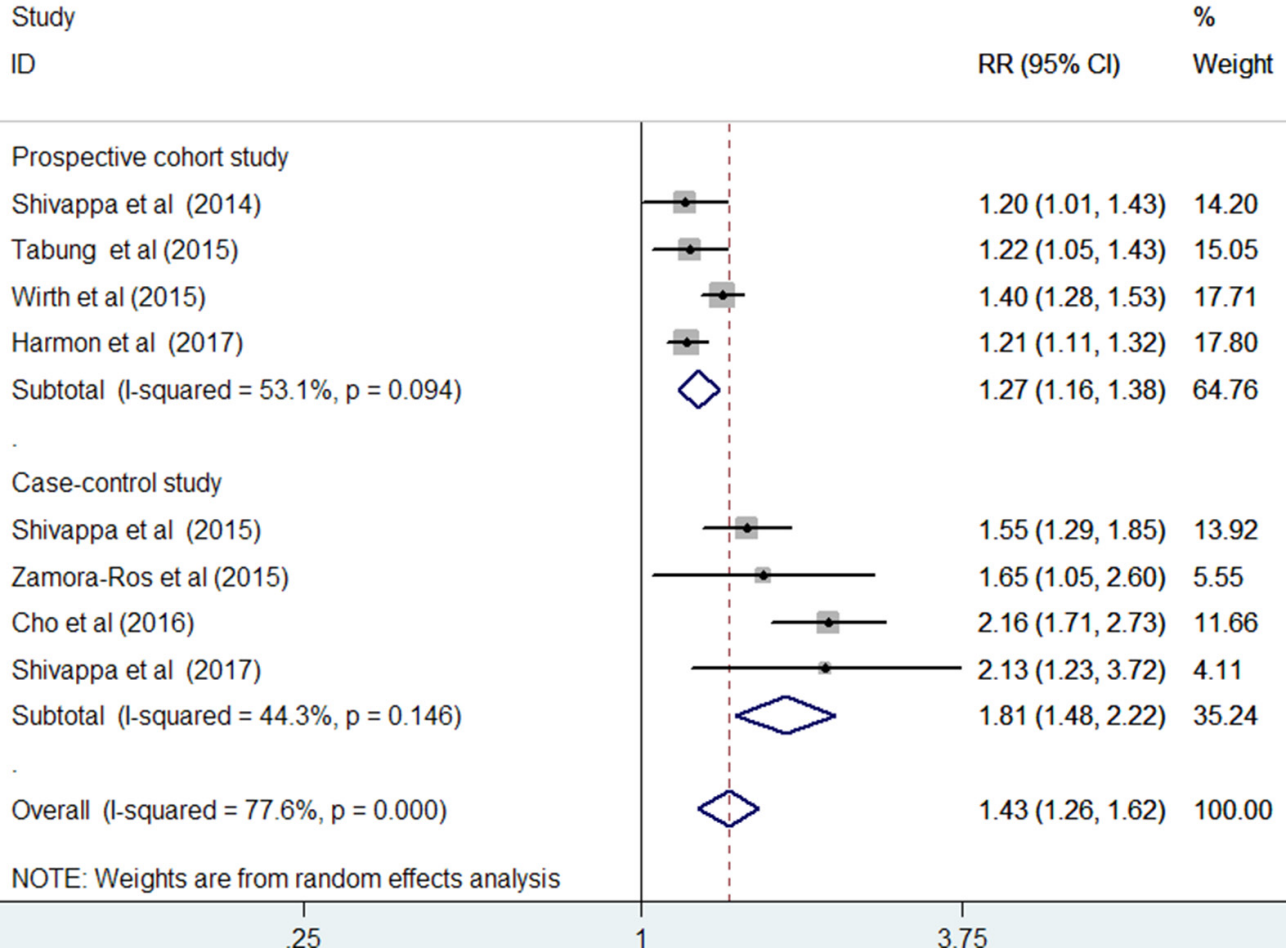
Forest plots showing RR with 95% CI of colorectal cancer comparing the highest to the lowest dietary inflammatory index score

**Figure 3:**
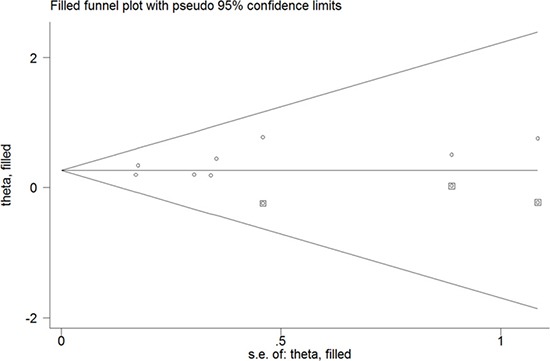
Funnel plot of dietary inflammatory index score with colorectal cancer risk The circles alone are real studies and the circles enclosed in boxes are ‘filled’ studies.

All the included studies investigated the association of DII score with CRC risk. The pooled RR for the highest versus lowest DII score was 1.43 (95% CI: 1.26–1.62) under a random-effect model (Figure 2). Substantial heterogeneity (I^2^ =77.6%, *p* < 0.001) was also noted. Sensitivity analysis showed that any study only slightly affected the pooling effect size and revealed the reliability of our pooling summary. No evidence of publication bias was identified on the basis of Egger's test (*p* = 0.148) and Begg's test (*p* = 0.108). The trim-and-fill approach suggested three missing studies in the funnel plot (Figure 3). However, imputing these three potential missing studies did not alter the original significant association (RR = 1.30; 95% CI 1.08–1.57; *p* = 0.006). Results stratified by study design showed a stronger risk of higher DII score for CRC among the case–control studies (RR = 1.81; 95% CI: 1.48–2.22) than among the prospective cohort studies (HR = 1.27; 95% CI: 1.16–1.38).

### Gender-specific associations

**Figure 4:**
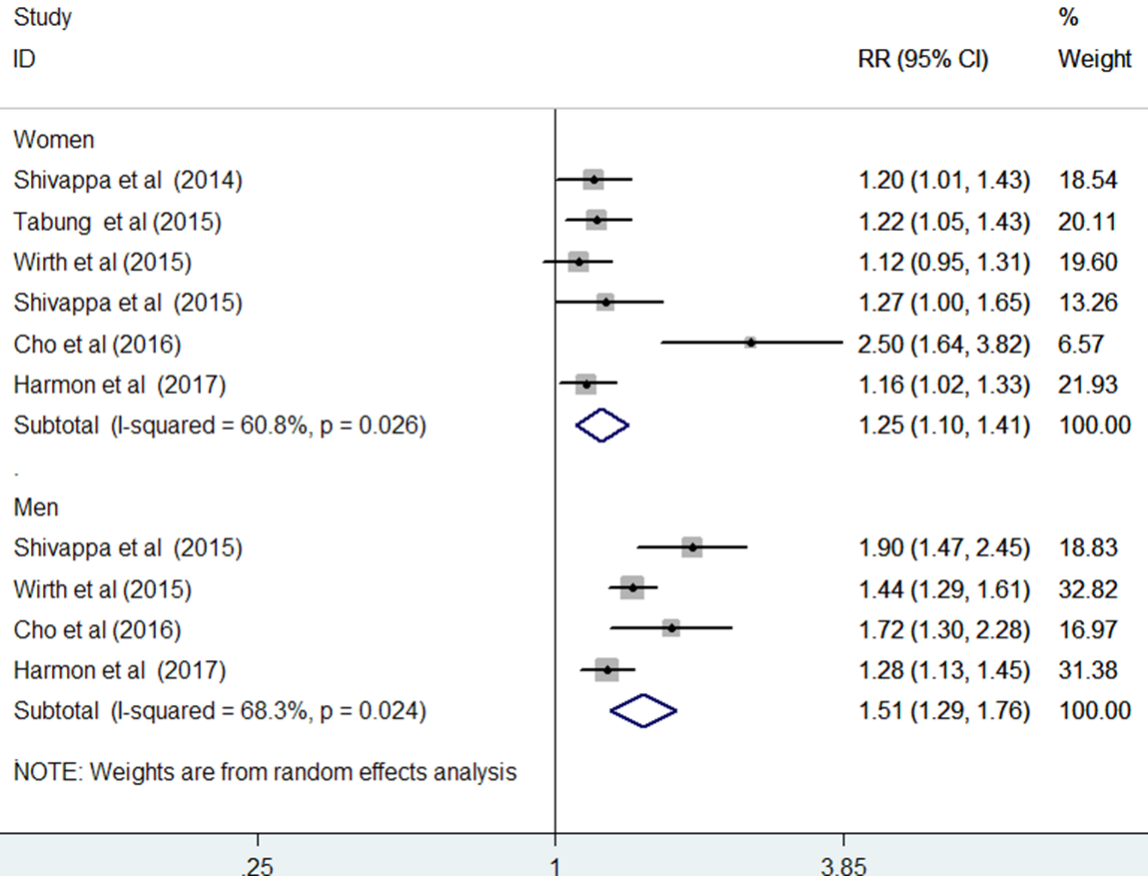
Forest plots showing gender specific RR with 95% CI of colorectal cancer comparing the highest to lowest dietary inflammatory index score

The results stratified by gender are shown in Figure 4. Four studies [13, 15, 17, 19] provided risk estimates by gender, whereas two studies [12, 14] reported risk estimates among women. When the highest DII scores were compared with the lowest scores, the pooled RR of CRC was 1.25 (95% CI: 1.10–1.41; I^2^ = 60.8%, *p* = 0.026) for women and 1.51 (95% CI: 1.29–1.76; I^2^ = 68.3%, *p* = 0.024) for men under a random-effect model.

### Cancer site-specific associations

**Figure 5:**
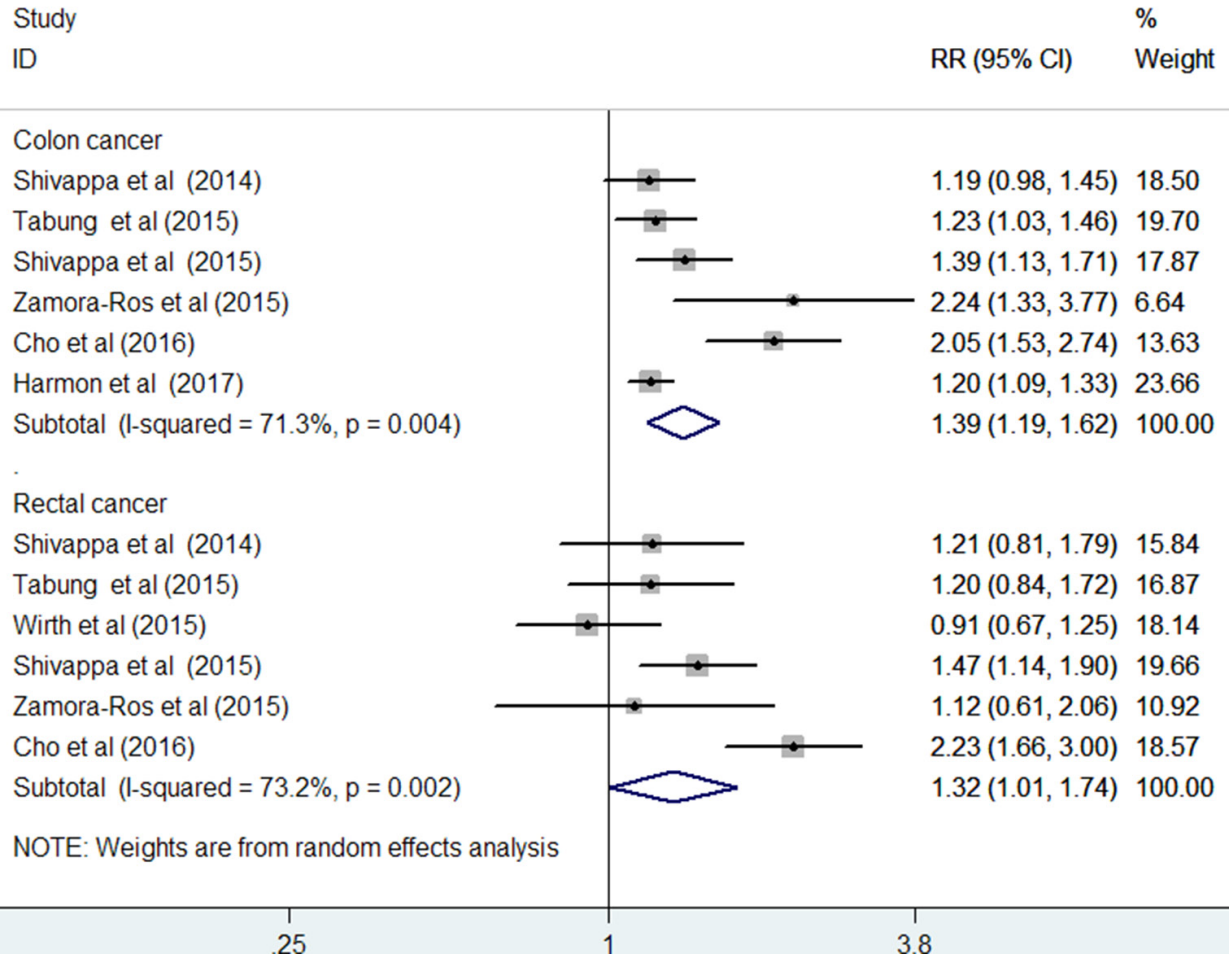
Forest plots showing RR with 95% CI of colon cancer and rectal cancer comparing the highest to lowest dietary inflammatory index score in all included studies

**Figure 6:**
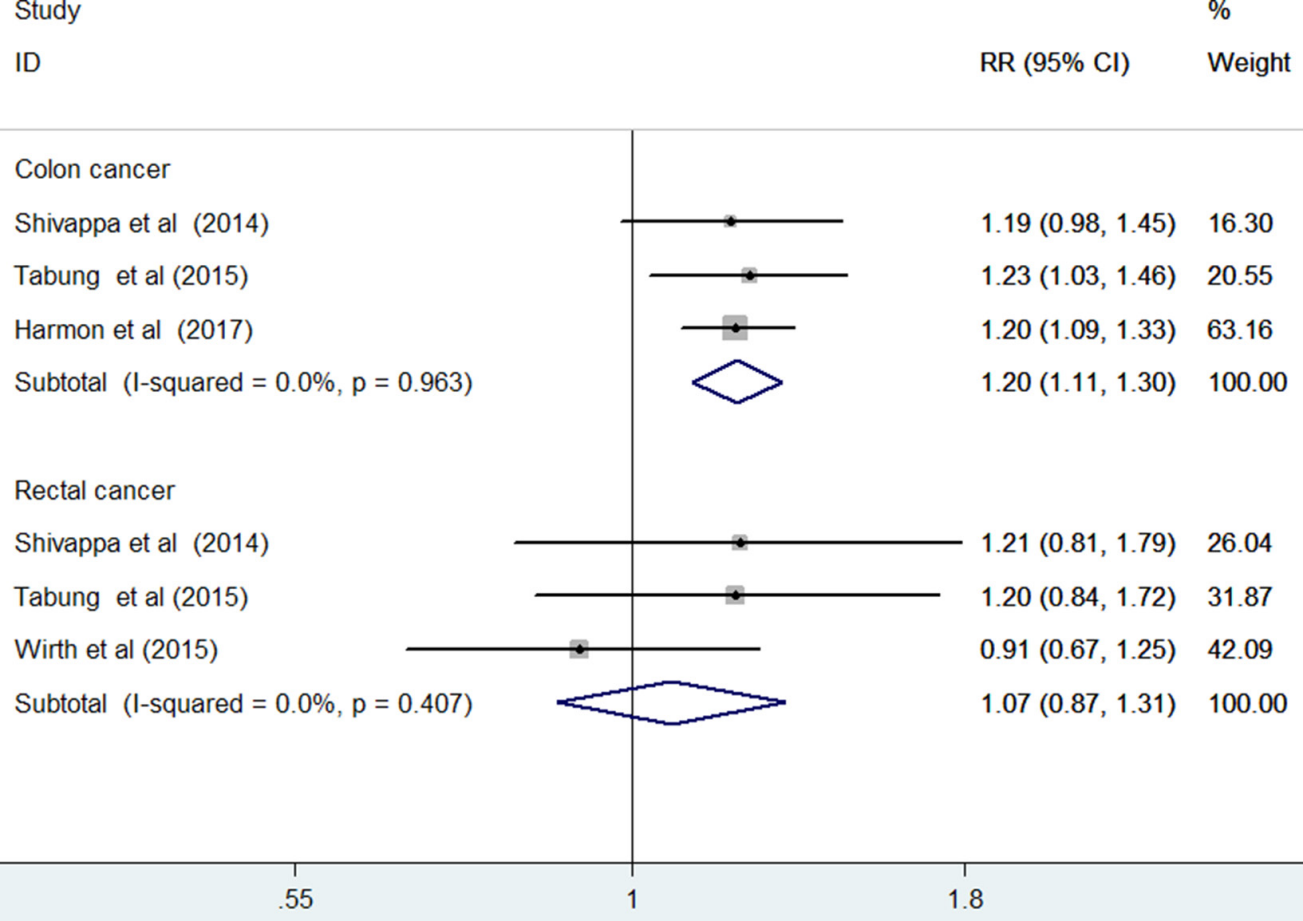
Forest plots showing RR with 95% CI of colon cancer and rectal cancer comparing the highest to lowest dietary inflammatory index score in prospective cohort studies

Seven studies [12–17, 19] reported the results stratified by cancer site. Compared with the highest to the lowest DII score, the pooled RR was 1.39 (95% CI: 1.19–1.62; I^2^ = 71.3%, *p* = 0.004) for colon cancer and 1.32 (95% CI: 1.01–1.74; I^2^ = 73.2%, *p* = 0.002) for rectal cancer in a random-effect model (Figure 5). Sensitivity analysis demonstrated the reliability of the pooled risk estimates for colon cancer. However, the pooled risk estimate for rectal cancer was not robust in the sensitivity analysis because of leaving out one study at each turn. Furthermore, when we restricted the analysis to the prospective cohort studies [12, 14, 15, 19] (Figure 6), the pooled RR was 1.20 (95% CI: 1.11–1.20) for colon cancer and 1.07 (95% CI: 0.87–1.31) for rectal cancer in a fixed-effect model.

## DISCUSSION

This meta-analysis provides evidence that a pro-inflammatory diet estimated by a high DII score is independently associated with increased CRC risk, especially colon cancer risk. Overall, individuals with maximal pro-inflammatory diet intake (highest DII score) showed a 43% increased CRC risk. This finding supports the notion that low inflammatory potential diets reduce CRC risk.

In our subgroup analyses, a more significant association of DII score with CRC risk was noted from the case–control studies than from the prospective cohort studies. However, case–control studies are susceptible to recall or selection bias; hence, additional prospective cohort studies are needed to confirm this finding. When the results were stratified by gender, the association between DII score with CRC risk tended to be stronger in men than in women. The mechanisms underlying the weaker association of DII score with CRC risk in women than in men were unclear.

CRC is a heterogeneous disease that occurs in the colon and the rectum. When we analyzed the cancer site-specific associations, higher DII score was independently associated with increased risks of colon cancer and rectal cancer in the overall analysis. However, our sensitivity analysis revealed that the association of DII score with rectal cancer risk was not reliable. In particular, rectal cancer showed no significant association with DII score when the analysis was restricted to the prospective cohort studies. This result suggests that the significant association depended on the results of the case–control studies. Given the lower reliability of case–control studies than cohort studies, the positive association of the DII score with rectal cancer risk may not be robust. The difference between the DII scores on colon cancer and rectal cancer risks may be explained by the different etiologies of colon and rectal cancers [20].

The DII score is a new dietary quality index that specifically focuses on the dietary inflammatory potential [11]. A higher DII score indicates a more pro-inflammatory diet, whereas a lower DII score signifies a more anti-inflammatory diet. In practice, the DII score is usually computed from dietary intake assessed using a validated FFQ or from historical dietary records. Apart from the significant association of the DII score with CRC risk, the DII score could also be used to predict the prolonged hospitalization and survival among CRC patients treated surgically [21, 22]. However, no statistically significant associations were found between a pro-inflammatory diet and the risk of colorectal adenoma recurrence [23]. Increased insulin resistance caused by systemic inflammation promoted by pro-inflammatory diets may be linked to the association between the DII score and CRC risk [24].

This study holds important implications for clinical practice. Our meta-analysis suggests that consuming pro-inflammatory diet, as estimated by a high DII score, is associated with increased CRC risk. With diet as a modifiable factor, limiting pro-inflammatory diets and/or favoring anti-inflammatory diets may be a strategy to reduce CRC risk. However, the present study results are unclear on the reliability of the cancer site-specific associations with the DII score. Further prospective studies on the cancer site-specific associations are warranted. To create a healthy diet, several anti-inflammatory foods, including fruits, vegetables, fish or fish oil, walnuts, brown rice, and bulgur wheat, should be included in the diet [25]. Moreover, refined or processed foods should be avoided, and red meat or full-fat dairy foods should be consumed less frequently.

Several limitations should be noted in this meta-analysis. First, the DII score was computed by self-report from the FFQs or historical dietary records, which carried an inherent recall bias. Second, the DII score was estimated at baseline, and potential changes in dietary habits during the follow-up duration could not be excluded. However, adult dietary habits tend to be relatively stable over time [26, 27]. Third, significant heterogeneity was found among the pooled studies. The differences in food items considered in the DII score, demographic characteristics, and cancer site may contribute to the observed heterogeneity. Fourth, potential publication bias might have occurred because our meta-analysis was based on a small number of studies. However, the trim-and-fill method indicated that potentially missing studies did not alter the significant original association and suggested the robustness of the results against publication bias. Finally, most study participants were of European descent. Therefore, generalizing these findings to diverse populations should be taken with caution.

In conclusion, this meta-analysis suggests that pro-inflammatory diet, as estimated by the DII score, is independently associated with increased CRC risk. These findings highlight the need to promote healthy dietary patterns with minimal inflammatory potential to reduce CRC risk. However, gender- and cancer site-specific associations need to be further investigated in future well-designed prospective studies.

## MATERIALS AND METHODS

### Data sources and searches

We performed this meta-analysis in accordance with the guidelines of the Meta-Analysis of Observational Studies in Epidemiology [28]. Two authors (Y Fan and X Jin) independently searched the PubMed and Embase from inception to February 2017. Search terms included (inflammatory potential of diet OR dietary inflammatory index OR anti-inflammatory diet OR pro-inflammatory diet) AND (colon cancer OR rectal cancer OR CRC OR colorectal adenoma OR colorectal neoplasm) AND (cohort OR case–control OR epidemiologic OR follow-up). A manual search of the reference lists of the retrieved studies was also performed to identify any additional study.

### Study selection

Studies meeting the following inclusion criteria were included: 1) all cohort and case–control studies that reported on the association of the inflammatory potential of diets, as estimated by DII score, with CRC risk and 2) those that provided the multivariable-adjusted RR, hazard ratio (HR), or odds ratio (OR) with corresponding 95% confidence intervals (CI) of CRC for the highest DII score (highest pro-inflammatory diets) versus the lowest DII score (lowest pro-inflammatory diets).

### Data extraction and quality assessment

Data extraction and quality assessment were independently performed by two authors (Y Fan and X Jin). Any discrepancy between two authors was resolved through consensus. The following data were collected from each study: last name of the first author, publication year, geographical region, study design, sample sizes, number of cases/controls, source of controls (for case–control studies), proportion of women, age range or mean age, method of diet assessment, comparison of DII score, most fully adjusted risk estimate, duration of follow-up (for cohort studies), and adjustment for confounding factors in the statistical analysis. The methodological quality of the included studies was evaluated using a nine-star NOS [29]. This scale judges a study quality based on the selection, comparability, and ascertaining of outcome. A study achieving seven or more stars was considered to be of high quality.

### Statistical analyses

To assess the association of the DII score with CRC risk, we pooled the most fully adjusted risk estimate for the highest versus the lowest DII score. CRC is relatively rare; thus, OR was an approximate estimation for RR. Heterogeneity among studies was evaluated using the Cochrane Q and I^2^ statistic. A *p*-value for Cochrane Q < 0.10 or a I^2^ statistic > 50% indicated substantial heterogeneity [30]. A random-effect model (DerSimonian and Laird) was selected to calculate the summary effect in case statistical heterogeneity was observed; otherwise, a fixed-effect model was selected. Subgroup analyses were performed by study design, gender, and cancer site. Publication bias was assessed using the Begg's test [31], and Egger's test [32]. In addition, the trim-and-fill method was used to assess the possible influence of publication bias. A sensitivity analysis was conducted by removing individual studies at a time to analyze the robustness of the pooling risk estimate. All analyses were performed by using STATA 12.0 (Stata Corporation, College Station, Texas, USA).
